# An algorithm for computing profile likelihood based pointwise confidence intervals for nonlinear dose-response models

**DOI:** 10.1371/journal.pone.0210953

**Published:** 2019-01-25

**Authors:** Xiaowei Ren, Jielai Xia

**Affiliations:** 1 Department of Health Statistics, Fourth Military Medical University, Xi’an, Shaanxi, China; 2 Statistics and Data Insights, Bayer Healthcare Company Limited, Beijing, China; University of Copenhagen, DENMARK

## Abstract

This study was inspired by the need to estimate pointwise confidence intervals (CIs) for a nonlinear dose-response model from a dose-finding clinical trial. Profile likelihood based CI for a nonlinear dose response model is often recommended. However, it is still not commonly used in dose-finding studies because it cannot generally be calculated explicitly. Most previous research has mainly focused on the performance of the profile likelihood based CI method compared with other common approaches. However, there are still no reports on computing profile likelihood based pointwise CIs for an entire dose-response curve. Based on a previous dose-finding trial with binary-response data, this present study proposed to calculate profile likelihood based pointwise CIs by using the bisection method with proper calculation order for doses in the curve plus crude search when the expected response is close to a boundary. The convergence could be improved by applying appropriate starting values for the optimization procedure with straightforward programming techniques. The algorithm worked well in most cases based on the simulation study and it can be applied more generally to other dose-response models, and other type of data like normally distributed response. It would indeed be great to be able to use profile likelihood approaches more routinely when constructing pointwise CIs for nonlinear dose-response models.

## Introduction

In dose-finding studies, together with parameter estimates from the fitted dose-response model, the pointwise confidence intervals (CIs) of the expected response for doses on the entire curve that characterize the uncertainty of the fitted model are always needed to provide essential information for identifying the optimal dose (s). To compute CIs for parameters, the Wald-type CI is the most commonly used approximate CI mainly because of its intuitive appeal and computational ease. However, the use of this approach in nonlinear dose-response models and binomial inferences has been discouraged because of its woeful performance [1–5]. The profile likelihood approach is one of the recommended methods for generating CIs for parameters from a nonlinear dose-response model [3–5]. Compared with Wald-type CI, the profile likelihood based CI generally has a better coverage, can avoid aberrations such as limits outside [0,1], and takes monotonicity into account. Furthermore, it even performs better than the bootstrap method (nonparametric percentile method) in some situations, such as when boundaries are used in the estimation of parameters in a nonlinear model [3–5].

However, profile likelihood based CI generally cannot be calculated explicitly, and is even more difficult to calculate when coping with two or more parameters in a nonlinear model [1,6,7]. Non-convergence occurs frequently when generating profile likelihood based CI through calculations by iteratively optimizing parameters, which is the most common cause of the calculation’s breakdown. The calculation can become more challenging when generating pointwise CIs for a large number of dose-points on a highly flexible nonlinear model [1,7]. This could be the reason why profile likelihood based pointwise CI is not commonly used for nonlinear dose-response models in clinical trials. Previous research has mainly focused on evaluating the performance of the profile likelihood approach for nonlinear dose-response models compared with other approaches [3–5,8]. However, there are to date no reports on computing pointwise CIs for an entire dose-response curve based on the profile likelihood approach. Considering the significant advantages of the profile likelihood based CI compared with other approaches [3–5], we aimed in the present study to investigate the implementation of profile likelihood based pointwise CIs for nonlinear dose-response models.

In this paper, we first discuss the issues when implementing the profile likelihood approach based pointwise CIs with an example from clinicaltrials.gov that has a binary efficacy endpoint in dose-response analysis. Subsequently, we outline the feasible methods to deal with the issues from an implementation point of view, and then a simulation study is performed to test the algorithm in different sample sizes and to evaluate the performance of profile likelihood based CIs for all doses for the entire curve compared with two other commonly used methods (Wald and Bootstrap approach based on nonparametric percentile method). Finally, conclusion and its discussion are presented.

## The motivation

The data that motivated this present study and that were used to illustrate the proposed analysis were extracted from trial NCT02131662 on clinicaltrials.gov. The trial was a phase 2 dose-finding study that was randomized and placebo-controlled with a total of four active doses (0.5 mg, 1 mg, 2 mg, and 4 mg) and placebo. The primary variable was assumed to be binomially distributed; the number of responders (n)/total number of participants (N) in each group from the lowest dose (0 mg, placebo) group to the highest dose (4 mg) group were as follows n/N = 1/58, 18/60, 34/61, 33/61, and 36/60. The dose-response model (4-parameter logistic model) was applied to investigate the dose-response relationship, which was assumed to be a monotonically increasing function in dose.

We next describe the issues and how to obtain profile likelihood based pointwise CIs with relatively general approaches based on this dataset. The analyses presented here are *post-hoc* analyses. In addition, normal-based pointwise Wald-type CIs and bootstrap pointwise CIs based on nonparametric percentile method were constructed to provide a direct comparison with the profile likelihood approach.

The 4-parameter logistic model as a generalized nonlinear model was specified as previously described [9,10]:

$$f(d,p_{0},E_{max},{ED}_{50},\delta )=p_{0}+E_{max}/\left\{1+e^{[{(ED}_{50}-d)/\delta ]}\right\},$$ (1)

Where *p*_0_ is the basal effect for *d* (dose) equal to -infinity, *E*_max_ is the maximum effect attributable to the drug (compared with the basal effect, the maximum increase of drug effect), *ED*_50_ is the dose at which 50% of *E*_max_ is achieved, and δ is the hill slope parameter controlling the rate of dose-dependent change in the effect. The estimation for the 4-parameter logistic model is based on maximum likelihood assuming binomial distributions. The 4-parameter logistic model is expected to have more flexible settings in dose-response analysis for binomially distributed data compared with 2-parameter logistic models, because it generalizes the usual logistic model to allow the lower and upper response asymptotes to be greater than zero and less than one, respectively. For example, the placebo effect result is a response greater than zero in the placebo group, or not all participants have the expected response to a drug [11]. This is the general case expected in clinical trials. However, this flexibility is at the cost of a higher dimensional optimization and makes harder to compute.

The commonly used algorithms for computing profile likelihood based CI include the one proposed by Venzon and Moolgavkar which is based on a modified Newton-Raphson iteration [7], the bisection method, and grid search [3,12]. For the algorithm proposed by Venzon and Moolgavkar, it has been noted that non-convergence might occur when assumptions are not met, including when the gradient at the maximum likelihood (ML) estimation deviates from zero, or when the likelihood is not close to a quadratic form, thus making the initial value of the iteration algorithm too small or too large [12]; This becomes even more challenging when fitting a nonlinear curve [7]. This algorithm was applied in our present example, but the non-convergence issue halted the implementation when handling four parameters and computing pointwise CIs for the entire curve from this highly flexible nonlinear model, which could not be fixed by simple programming techniques. Therefore, methods for general purposes with relative straightforward techniques, i.e., bisection method plus crude search, were considered and tested in order to minimize non-convergence readily by applying the appropriate initial values for iteration with simple programming techniques. All information needed for the iteration can be taken directly from the output from a previous fitting and no extra efforts were required to calculate their derivatives with these methods. Indeed, this has made the calculation very easy when computing the pointwise CIs.

## The algorithm for computing pointwise CIs

To obtain the pointwise CIs, a grid of doses ranging from the lowest dose to the highest dose (0–4 mg) was defined. A total of 41 equidistant points, d* (0–4 mg, by 0.1 mg), were defined in our example. The grid of values can be extended to obtain greater accuracy, if necessary. In addition, we reparameterized the model to encompass our target parameter which is the expected response p* in our case. We replaced *E*_max_ with a function of p* in model (1),

$$E_{max}=(p^{*}-p_{0})\{1+e^{[{(ED}_{50}-d^{*})/\delta ]}\}$$

thus making the model contain p*

$$f(d,p_{0},p^{*},d^{*},\delta ){=p}_{0}+\left(p^{*}-p_{0})\{1+e^{[{(ED}_{50}-d^{*})/\delta ]}\right\}/\left\{1+e^{[{(ED}_{50}-d)/\delta ]}\right\}$$ (2)

This is in principle the usual approach for estimating confidence intervals of a certain estimate [3,4,8]. After the reparameterization, the profile likelihood *versus* the expected response can be obtained in the model [8], and subsequently to obtain the CI of p* for each specific dose d* in the defined grid like other parameters.

### Bisection method

We started the implementation of the bisection method on our example. The bisection method is a simple and robust root-finding method. In our example, the search algorithm stopped when the difference between the upper and lower bound of the search interval was less than the convergence tolerance set in advance. The midpoint of the next-to-last search interval was taken as the lower or upper confidence limit. The greater accuracy can be obtained by setting smaller convergence tolerances.

A reasonable lower and upper bound of the initial search interval can speed up the convergence in bisection method. The natural upper boundary for binomial data is 100%, which could be used as the upper bound of the initial search interval in the calculation of the upper confidence limit for each dose. However, as dose-response models are often monotone, for monotone models, if the model fits significantly better than the constant model, for example, based on a likelihood ratio test, the profile likelihood based confidence bounds will be monotone [4]. Because the pointwise CIs can be estimated sequentially, the monotonicity of confidence limits can be taken into account to facilitate the calculation when setting the lower and upper bound of the initial search interval of a dose. Thus, for a specific dose apart from the highest dose in the dose grid defined above, the already calculated upper confidence limit for the expected response of the immediate dose that is higher to this specific dose can be a good alternative to the upper bound of the initial search interval for its upper confidence limit. Therefore, we started the calculation for upper confidence limits from the highest dose. In our case, the highest dose was 4 mg, and the upper bound of the initial search interval for the upper confidence limit of the expected response for this dose was set as $\hat{p}+5se(\hat{p})$, where $\hat{p}$ is the ML estimate of the expected response to 4 mg and $se(\hat{p})$ is the standard error of the expected response to 4 mg. In the simulation study (the simulation section), there were no upper confidence limits of the expected response to 4 mg that were larger than $\hat{p}+3.5se(\hat{p})$ in all the tested sample sizes. The maximum multiple of $se(\hat{p})$ for different sample sizes based on profile likelihood CIs from the simulation study for our example can be found in Table 1. Thus, the value used as the upper bound of the initial search interval for 4 mg was a reasonable value which was large enough to cover the upper confidence limit of the expected response to 4 mg. In our case, this value is less than 100%. However, if this value is higher than 100% due to a very high expected response of the highest dose, 100% should be used as the upper bound of the initial search interval for the highest dose for binomial data. A reasonable lower bound of the initial search interval for the upper confidence limit of the expected response for each dose can be its ML estimate. A similar approach was implemented to calculate the lower pointwise confidence limits of the curve, except that the calculations were started from the lowest dose. Therefore, for a specific dose apart from the lowest dose in the dose grid, the already calculated lower confidence limit for the expected response of the immediate dose lower to this dose can be used as the lower bound of the initial search interval for its lower confidence limit. The lowest dose in our case was 0 mg. To simplify the calculation the lower bound of the initial search interval for the lower confidence limit of 0 mg dose was set at 0%. Based on the simulation study (the simulation section), there were no lower confidence limits of the expected response to 0 mg that were less than $\hat{p}‑2se(\hat{p})$ in all the tested sample sizes, which is a value very close to 0 in our case. Similarly, the ML estimate of the expected response for each dose can be used as the upper bound of the initial search interval for the lower confidence limit of this dose. In addition, the ML estimates for all parameters from a previous fitting were used as the starting values of the parameters for the next optimization.

**Table 1:** Maximum multiple of $se(\hat{p})$ for dose 0 mg and 4 mg based on profile likelihood confidence intervals for different sample sizes from the simulation study.

	Maximum multiple of $se(\hat{p})$^a^ for lower limit		Maximum multiple of $se(\hat{p})$^a^ for upper limit	
n	0 mg	4 mg	0 mg	4 mg
20	1.4	2.1	16.6	3.3
50	1.8	2.7	10.3	3.2
70	1.9	1.8	11.6	2.4
100	1.8	1.6	8.7	1.8

^a^ $\hat{p}$ is the maximum likelihood estimate of the expected response rate; $se(\hat{p})$ is the standard error of the expected response rate. $se(\hat{p})$ is computed based on approximate normality.

An alternative feasible solution is to start the calculation from the lowest dose for the upper confidence limits and from the highest dose for the lower confidence limits. For example, when calculating pointwise upper confidence limits for a specific dose, the already calculated upper confidence limit for the expected response of the immediate dose lower to this dose can be used as the lower bound of the initial search interval for its upper confidence limit. In this way, a reasonable upper bound of the initial search interval for the upper limit for a dose is needed to be set. However, we have met some problems with our example when handling doses at or close to 0 mg which is described in next section, therefore we started the calculation from the highest dose for the upper confidence limits.

### Crude search

With the above-mentioned approach, non-convergence did not occur in the calculation for most doses in our example, which worked well except in those doses with a very low expected response rate (close to 0%). In our example, this issue was more relevant to the upper confidence limits calculation for doses at or close to 0 mg. It is well known that suitable starting values of parameters are needed to ensure convergence of the estimation algorithm when reparameterizing nonlinear regression models [13,14]. In our case, ML estimates from the nearby fitting could be the suitable starting values for a specific fitting. To implement this, a crude search for the confidence limit starting from the ML estimate of the expected response of a dose was considered. In this way, we could easily take the ML estimates of three nuisance parameters from the immediate previous fitting as the starting values for the next fitting. The search stopped when the profile log-likelihood value of the fitting was lower than the threshold $l(\hat{p})-0.5\chi_{1}^{2}(0.95),$ where *l*(.) is the log likelihood function and $\chi_{1}^{2}(0.95)$ is the 0.95 quantile of a chi-squared distribution with one degree of freedom. The non-convergence can be avoided to a large degree in this way; if not, smaller search-steps can be applied until no convergence issue occurs. With few attempts in our example, searching with a step increase of 1% was used when the profile log-likelihood value was far from the threshold, and an increase of 0.1% was used when the searching was close to the threshold. If needed, refining the searching steps around the upper limit can be applied to get greater accuracy. For doses higher than 0 mg, the search can start from the upper confidence limit of the 0 mg dose or of a corresponding lower nearby dose. In our example, the crude search was applied for doses lower than 0.3 mg. As mentioned, this issue occurred more often when calculating the upper limits for doses at or close to 0 mg in our example. However, if the same issue occurs for the lower limits calculation, the same method can be applied. Although this method made the calculations take longer in standard cases, it worked well and appeared necessary for doses at or close to 0 mg.

### Results

**Fig 1:**
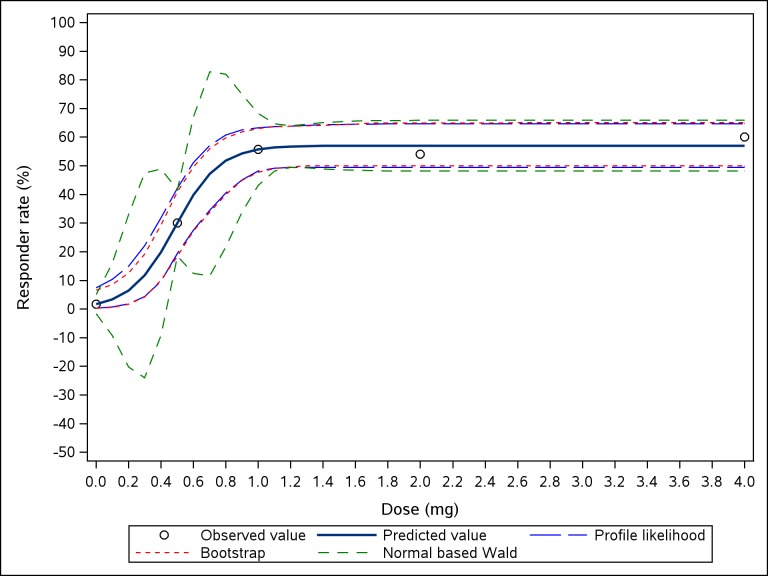
Pointwise confidence intervals for a 4-parameter logistic model. Three different methods are shown to estimate confidence intervals.

The profile likelihood based pointwise CIs are shown in Fig 1. To give a direct comparison, normal-based pointwise Wald-type CIs and bootstrap pointwise CIs based on nonparametric percentile method are also provided (Fig 1).

The disadvantages of the normal-based pointwise Wald-type CIs were obvious in our example, which has been also discussed in previous reports as follows: the lower confidence limits of a dose nearby placebo are negative [2,4]; pointwise CIs are non-monotone as function of dose [4]; coverage rates are not correct for some doses [3,4,15], especially for doses between two observed doses. The non-monotone lower or upper limits by Wald approach partly depend on the experimental design and the chosen model. We tested this with our data in a different dose-response model: *E*_max_ model and also tested this with a better dose design (simulated data): 0.7 mg group was added between 0.5 mg and 1 mg groups. The Wald-type CIs were monotone in both scenarios.

Bootstrap pointwise CIs have been computed based on the nonparametric percentile method, which is a good approach to construct CIs especially for nonlinear dose-response models [3,16,17]. In our example, the results from the bootstrap method were similar to those from the profile likelihood approach (Fig 1).

Computation of the profile likelihood based pointwise CIs with the algorithm we proposed was performed with SAS (version 9.2). SAS macros for bisection method were developed based on the macros called ‘Plkhci’ and ‘BinomialProfile’ defined in Millar R’s book [6]. The estimation for the 4-parameter logistic model was obtained using PROC NLMIXED with assuming binomial distributions. The full SAS code based on our example is provided in S1 Appendix. Boundaries were used to improve convergence, which are described in S1 Appendix. Wald-type CIs were obtained using PROC NLMIXED directly [18]. The resampling for bootstrap was performed with PROC SURVEYSELECT in SAS (version 9.2) with 1,000 bootstrap samples [16,19].

The code can be easily adjusted and used for other type of dose-response models. We tested the proposed algorithm with the data example (normally distributed response) used in the paper by Baayena and Hougaard [4] to calculate the profile likelihood based pointwise CIs for the full curve and the effect curve fitted with *E*_max_ model. The dataset for this example is available from the R package DoseFinding [20]. The proposed algorithm worked well and the pointwise intervals were almost identical to those showed on the figures provided in the paper by Baayena and Hougaard. The code for this normally distributed response example is similar and obtainable upon request.

## A simulation study

To test the algorithm proposed in different sample sizes and to evaluate the performance of the profile likelihood approach in different doses for the entire curve, a simulation study was performed. The simulation study was based on the example discussed in the motivation section with the following doses (0 mg, 0.5 mg, 1 mg, 2 mg, and 4 mg) in a simulated 4-parameter logistic model. The coefficients were chosen to be the ML estimates based on our example (*p*_0_ = 0.15%, *E*_max_ = 56.9%, *ED*_50_ = 0.49 mg, δ = 0.14). Sample sizes of 20, 50, 70, and 100 per dose-group were generated. For each sample size, we simulated 1,000 datasets. For each set of data, the profile likelihood, normal-based Wald, and nonparametric percentile bootstrap pointwise 95% CIs were calculated. For the nonparametric percentile bootstrap, 1,000 resamples were generated. Simulation study was written in SAS (version 9.2).

For the calculations with medium and relatively large sample sizes (n = 50, 70, or 100), the profile likelihood based pointwise CIs with the approaches defined above worked generally well. However, the non-convergence happened more frequently in the calculation for the small sample size (n = 20); thus, crude searches for more doses were applied. To reduce the efforts spent on debugging, a higher dose for using crude search was set in the simulation, as crude search is always a workable approach. In the simulation for the sample size of 20, the profile likelihood based pointwise CIs were constructed successfully for 99.8% of the datasets, and pointwise CIs in two datasets were not constructed successfully based on the logistic model due to a U-shape curve expected from the resamples. It was rather difficult to obtain convergence with the defined logistic dose-response model. For the other sample sizes, pointwise CIs were constructed for all datasets without failure.

**Fig 2:**
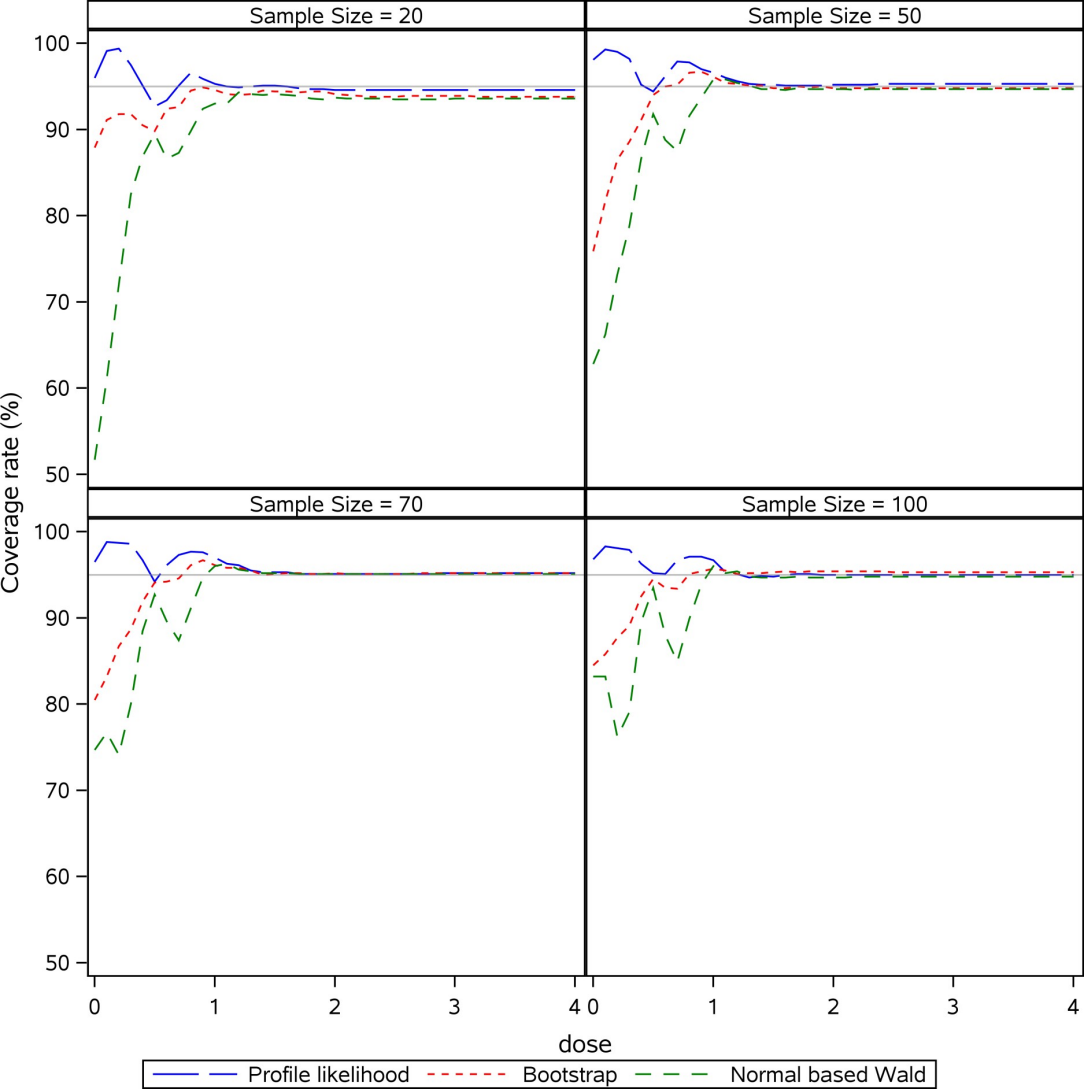
Coverage rates of confidence intervals for each dose-point in a 4-parameter logistic curve. Three different methods are shown to estimate the coverage rates.

In addition, we evaluated the coverage rates for the three common approaches in different doses simultaneously via the simulation study (Fig 2). For all approaches and all sample sizes tested, the coverage rates were very close to the nominal level for doses in or close to plateau (in our case, the doses were higher than 1 mg). For doses around or below *ED*_50_(~0.5 mg), Wald-type and nonparametric percentile bootstrap performed erratically, but the coverage rates were generally lower than the nominal level. Especially for the small sample sizes, the coverage rates of these two approaches were constantly lower than the nominal level. However, profile likelihood based pointwise CIs were much closer to the nominal level and were superior to all other methods for those low doses, even in small sample sizes.

## Discussion and conclusion

In this study, we have addressed the computation of profile likelihood based pointwise CIs for a nonlinear 4-parameter logistic model with binomially distributed data based on a phase 2 dose-finding trial. An algorithm, the bisection method with proper calculation order for doses in the curve, plus crude search when the expected response was close to a boundary, was proposed. The starting values for a specific fitting with the ML estimates of parameters from previous fitting and the log likelihood value for each fitting can be taken from the standard output of the SAS procedure with straightforward programming techniques, with no extra efforts. The non-convergence can be minimized significantly with this proposed approach.

In the simulation study, we applied and tested this algorithm in different sample sizes. They were relatively easy to use or implement and worked well in most cases. However, for small sample sizes, the calculation failed for very few datasets due to odd resampling data. In practice, this could be avoided by defining a more appropriate dose-response model based on observed data. In addition, in our simulation study, we observed that the coverage rates of profile likelihood based pointwise CIs were closer to the nominal level than the other approaches, including the bootstrap method, for almost all doses, especially for doses with an expected response rate close to 0%. The good performance of the profile likelihood approach was consistent in each tested sample size including the small sample size. Our example and the simulation study both confirmed that Wald-type pointwise CIs perform badly in nonlinear models, including non-monotone CIs, yielding an unreasonably lower confidence limit of the expected response to placebo and incorrect coverage rates for some doses [2–4].

We also conclude that the crude search is relatively time-consuming and might make the calculation take longer in standard cases. The profile likelihood pointwise confidence intervals for the entire curve were computed in our example in approximately a minute and a half which is comparable to a bootstrap approach with 2,000 bootstrap samples. Nevertheless, the algorithm as proposed in this present study is essentially workable and appears to be necessary especially for doses with response rates close to 0% when facing highly flexible nonlinear models. The non-convergence can be minimized by straightforward program techniques, which may motivate people to apply this approach in this field. In addition, considering the ongoing increasing speed of the computer systems, the speed of calculation should not be a limiting factor for not using this simple and robust algorithm, especially when handling nonlinear models requiring intensive computations. We tested the algorithm in an example with normally distributed response fitted with *E*_max_ model. The code provided in S1 Appendix can be easily changed and used. Therefore, although we only discussed the issues and application for a 4-parameter logistic dose-response model with binomially distributed data, the same considerations could be applied in other dose-response models with different type of data. This algorithm can be applied more generally.

Overall, from a practical point of view, we believe that the implementation of this relatively straightforward algorithm can promote the application of the profile likelihood approach for constructing pointwise CIs for nonlinear dose-response models.

## Supporting information

S1 Appendix SAS program to compute profile likelihood based pointwise confidence intervals. (DOCX)
