# Supplementary material for: An algorithm for computing profile likelihood based pointwise confidence intervals for nonlinear dose-response models
Source: PLoS One. 2019 Jan 25;14(1):e0210953. doi: 10.1371/journal.pone.0210953 (PMC6347253; doi:10.1371/journal.pone.0210953)
Supplement: S1 Appendix — (DOCX) [file pone.0210953.s001.docx]

*****************************************************************************************

This SAS program is to compute profile likelihood based pointwise confidence intervals

(CIs) for a 4-parameter logistic model with binary-response data.

The example data was extracted from clinicaltrials.gov.

*****************************************************************************************;

*Example data;

DATA ExpData;

INPUT dose respyes respno ntotal @@;

CARDS;

0 1 57 58

0.5 18 42 60

1 34 27 61

2 33 28 61

4 36 24 60

;

*****************************************************************************************

%BinomialProfile is to obtain the parameter estimates and the profile log-likelihood

value from each fitting.

%BinomialProfile is developed based on the same name macro in the book by Russell B.

Millar: "Maximum Likelihood Estimation and Inference. With examples in R, SAS and ADMB".

Reparameterized dose-response model is used.

Boundaries are used to improve convergence. Boundaries are set based on previous research.

Parameters:

p: Expected response at a specific dose

p1: Starting value for P0 (basal effect level for dose equal to -infinity)

p2: Starting value for ED50 (the dose at which 50% of Emax is achieved)

p3: Starting value for Delta (hill slope parameter)

ds: Dose from a grid of doses which is defined in %pwcl

*****************************************************************************************;

%MACRO BinomialProfile(p,p1,p2,p3);

PROC NLMIXED DATA=ExpData;

PARMS p0=&p1. ed50=&p2. delta=&p3.;

BOUNDS p0>=0, ed50>0, delta>0.1;

mu = p0+(&p.-p0)*((1 + EXP((ed50-&ds.)/delta))/(1 + EXP((ed50-dose)/delta)));

MODEL respyes~BINOMIAL(ntotal,mu);

RUN;

%MEND BinomialProfile;

*****************************************************************************************

%Plkhci is to compute profile likelihood based CI with bisection approach.

%Plkhci is developed based on the same name macro in the book by Russell B. Millar:

"Maximum Likelihood Estimation and Inference. With examples in R, SAS and ADMB".

Parameters:

macroname: Profiling macro name

lower: Lower bound of the initial search interval for the starting dose

For upper limit, it's '&est' from %pwcl

For lower limit, it's '&upp' from %pwcl

upper: Upper bound of the initial search interval for the starting dose

For upper limit, it's '&upp' from %pwcl

For lower limit, it's '&est' from %pwcl

thd: The threshold for l(p). It's from %pwcl

pest0: Starting value for P0

pest1: Starting value for ED50

pest2: Starting value for Delta

tol: Convergence tolerance.

*****************************************************************************************;

%MACRO Plkhci(macroname,lower,upper,thd,pest0,pest1,pest2,tol=0.00001);

* Define the starting values for P0, ED50 and Delta;

%GLOBAL pp0;

%GLOBAL ped50;

%GLOBAL pdelta;

%GLOBAL uplw;

%IF &side="U" AND &ds=4 %THEN %DO;

%LET _lower=%SYSEVALF(&lower);

%LET _upper=%SYSEVALF(&upper);

%end;

%ELSE %IF &side="U" %THEN %DO;

%LET _lower=%SYSEVALF(&lower);

%LET _upper=%SYSEVALF(&uplw);

%END;

%IF &side="L" AND &ds=0 %THEN %DO;

%LET _upper=%SYSEVALF(&upper);

%LET _lower=%SYSEVALF(&lower);

%END;

%ELSE %IF &side="L" %THEN %DO;

%LET _upper=%SYSEVALF(&upper);

%LET _lower=%SYSEVALF(&uplw);

%END;

%LET pp0=%SYSEVALF(&pest0); *P0;

%LET ped50=%SYSEVALF(&pest1); *ED50;

%LET pdelta=%SYSEVALF(&pest2);*Delta;

%LET ProfileIteration=0;

%DO %WHILE (%SYSEVALF(%sysfunc(abs(&_upper-&_lower))>&tol));

%LET profilepar=%SYSEVALF(0.5*(&_lower+&_upper));

%LET ProfileIteration=%EVAL(&ProfileIteration+1);

ODS OUTPUT FitStatistics=_FitStatistics;

ODS OUTPUT ParameterEstimates=_Estimates;

%&macroname(&profilepar,&pp0,&ped50,&pdelta);

DATA _null_;

SET _FitStatistics;

IF _N_=1;

target=%SYSEVALF(&thd);

LogL=-Value/2;

profilepar=%SYSEVALF(&profilepar);

IF ((&side="L" AND LogL>target)OR(&side="U" AND LogL<target)) THEN

CALL SYMPUT("_upper",profilepar);

ELSE

CALL SYMPUT("_lower",profilepar);

RUN;

%END;

*Take ML estimates for parameters from a previous fitting. These values will be used

in the next fitting as the starting values;

DATA _null_;

SET _Estimates;

IF parameter="p0" THEN CALL SYMPUT("pp0",estimate);

IF parameter="ed50" THEN CALL SYMPUT("ped50",estimate);

IF parameter="delta" THEN CALL SYMPUT("pdelta",estimate);

RUN;

*Create dataset: upper/lower limit for each dose in the grid.

For upper limits calculation, this upper limit for the expected response will be used

as the upper bound of the initial search interval for the next dose.

For lower limits calculation, this lower limit for the expected response will be used

as the lower bound of the initial search interval for the next dose.;

DATA ci&dsm.;

cl=&profilepar;

dose=&ds.;

CALL SYMPUT("uplw",cl);

RUN;

%MEND Plkhci;

*****************************************************************************************

%GlobalSearch is to perform a crude Search.

Parameters:

gmacroname: Profiling macro name

startv: Starting value for crude search

It is the ML estimate of the expected response of a dose and from %pwcl

gest0: Starting value for P0

gest1: Starting value for ED50

gest2: Starting value for Delta

llf: The maximized value of the log-likelihood for p of each dose.

It's from %pwcl

thd: The threshold for l(p) which is from %pwcl. It's from %pwcl

*****************************************************************************************;

%MACRO GlobalSearch(gmacroname,startv,gest0,gest1,gest2,llf,thd);

%GLOBAL gp0;

%GLOBAL ged50;

%GLOBAL gdelta;

%GLOBAL uppg;

%LET GProfileIteration=0;

%LET LogLm=%SYSEVALF(&llf);

%IF &ds.=0 %THEN %DO;

%LET gp0=%SYSEVALF(&gest0); *p0;

%LET ged50=%SYSEVALF(&gest1); *ed50;

%LET gdelta=%SYSEVALF(&gest2); *delta;

%LET profilepar=%SYSEVALF(&startv);

%END;

%ELSE

%LET profilepar=%SYSEVALF(&uppg);

%DO %UNTIL (%SYSEVALF(&LogLm<&thd));

%IF %SYSEVALF(&LogLm-&thd)<=1.6 %THEN

%LET profilepar=%SYSEVALF(&profilepar+0.001);

%ELSE

%LET profilepar=%SYSEVALF(&profilepar+0.01);

%LET GProfileIteration=%EVAL(&GProfileIteration.+1);

ODS OUTPUT FitStatistics=_GFitStatistics;

ODS OUTPUT ParameterEstimates=_GEstimates;

%&gmacroname(&profilepar,&gp0,&ged50,&gdelta);

DATA _null_;

SET _GFitStatistics;

IF _N_=1;

LogL=-Value/2;

CALL SYMPUT("loglm",logl);

gprofilepar=%SYSEVALF(&profilepar);

RUN;

DATA _null_;

SET _GEstimates;

IF parameter="p0" THEN CALL SYMPUT("gp0",estimate);

IF parameter="ed50" THEN CALL SYMPUT("ged50",estimate);

IF parameter="delta" THEN CALL SYMPUT("gdelta",estimate);

RUN;

%END;

DATA ci&dsm.;

cl=&profilepar;

dose=&ds.;

CALL SYMPUT("uppg",cl);

RUN;

%MEND GlobalSearch;

*****************************************************************************************

%pwcl is to compute pointwise CIs for entire dose-response curve.

Parameters:

side: U/L(Upper/Lower limit of CI)

start: Starting dose for the calculation

end: Ending dose for the calculation

step: Step in the grid of the doses

crude: N/Y crude search needed/ not needed.

****************************************************************************************;

%MACRO pwcl(side=U, start=,end=,step=,crude=N);

*To define the dose grid;

%DO dsm= &start. %TO &end. %BY &step.;

%LET ds=%SYSEVALF(&dsm./10); *dose used in the computation;

*The reparameterized model in the manuscript is applied.

The estimation for the 4-parameter logistic model is obtained using PROC NLMIXED with

assuming binomial distributions.

'pred' is the target parameter (expected response).

PROC NLMIXED is used to obtain:

1) Maximized value of the log-likelihood for p of each dose.

2) Starting values of the parameters for computing pointwise CIs.

The optimization is sensitive to the initial value for 'pred'.

Therefore different initial values are used for different dose range.

The convergence can be improved by this way.

These values are roughly estimated values and can be adjusted if necessary.;

ODS OUTPUT FitStatistics=_OFitStatistics;

ODS OUTPUT ParameterEstimates=_OEstimates;

PROC NLMIXED DATA=ExpData ;

PARMS p0=0 ed50=0.45 delta=0.2

%IF &ds.<0.3 %THEN pred=0.01;

%ELSE %IF &ds.<0.8 %THEN pred=0.3;

%ELSE %IF &ds.>=0.8 %THEN pred=0.5 ;;

BOUNDS ed50>0, delta>0.1, p0>=0;

mu = p0+(pred-p0)*((1 + EXP((ed50-&ds.)/delta))/(1 + EXP((ed50-dose)/delta)));

MODEL respyes~BINOMIAL(ntotal,mu);

RUN;

DATA _null_;

SET _OFitStatistics;

IF _N_=1;

*'NLLFREE' is the maximized value of the log-likelihood for p of each dose;

NllFREE=-Value/2;

CALL SYMPUT("NLogL",NllFREE);

*'thd' is the threshold for calculating profile likelihood based interval;

*the CI is given by all values of p for which l(p) should be > 'thd' value;

threshold =NllFREE-CINV(1-0.05,1)/2;

CALL SYMPUT("thd",threshold);

RUN;

DATA _null_;

SET _OEstimates;

IF parameter="pred" THEN CALL SYMPUT("EST",estimate);

%IF &ds=4 %THEN %DO;

percent=estimate+5*standarderror;

CALL SYMPUT("upp",percent);

%END;

%IF &ds=0 %THEN %DO;

percent=0;

CALL SYMPUT("low",percent);

%END;

IF parameter="p0" THEN CALL SYMPUT("est1",estimate);

IF parameter="ed50" THEN CALL SYMPUT("est2",estimate);

IF parameter="delta" THEN CALL SYMPUT("est3",estimate);

RUN;

%IF &crude=Y %THEN %DO;

%GlobalSearch(BinomialProfile,&est.,&est1.,&est2.,&est3.,&NLogL.,&thd.);

%END;

%ELSE %DO;

*call '%Plkhci' to compute pointwise CIs;

*upper limit;

%IF &side.="U" %THEN %Plkhci(BinomialProfile,&est.,&upp., &thd.,&est1.,&est2.,&est3.);

*lower limit;

%IF &side.="L" %THEN %Plkhci(BinomialProfile,&low.,&est., &thd.,&est1.,&est2.,&est3.);

%END;

%END;

%MEND pwcl;

*crude search;

%pwcl(start=0,end=4,step=1,crude=Y);

*compute upper limit;

%pwcl(side="U",start=40,end=5,step=-1);

*compute lower limit;

%pwcl(side="L",start=0,end=40,step=1);
